# Tempo-spatial dynamics of physicochemical properties and microbial communities in high-temperature Daqu during the fermentation process

**DOI:** 10.1016/j.fochx.2025.102815

**Published:** 2025-07-20

**Authors:** Yu-Hua Wei, Zhang Wen, Shang-Jie Yao, Ping-Yan Cheng, Wei Huang, Li—Li Jiang, Mei Bai, Da-Yong Han, Liang Song, Hai-Yan Zhu, Feng-Yan Bai, Di-Qiang Wang, Pei-Jie Han

**Affiliations:** a State Key Laboratory of Microbial Diversity and Innovative Utilization, Institute of Microbiology, Chinese Academy of Sciences, Beijing 100101, PR China; b College of Life Sciences, University of Chinese Academy of Sciences, Beijing 100049, PR China; c GuiZhou XiJiu Co., Ltd, Guizhou 564622, PR China; d College of Life Science, University of Hebei, Baoding 071002, PR China

**Keywords:** High-temperature Daqu, PacBio small-molecule real-time sequencing, Physicochemical properties, Metabolites, Microbial diversity

## Abstract

High-temperature Daqu (HTD) is the starter for sauce-flavor Baijiu, produced through stacked solid-state fermentation. At the end of fermentation, white, yellow, and black Daqu are typically formed in different layers of the stack, but the mechanisms behind their formation remain unclear. This study compared tempo-spatial dynamics of physicochemical properties, metabolites, and species-level microbial communities throughout HTD fermentation. Different layers of HTD remained similar during the initial five days of fermentation, but gradually exhibited significant physicochemical and microbial differentiation in the later stage (days 8–40). The upper-layer Daqu exhibited remarkably higher saccharification and liquefaction enzyme activities than the other layers. Thirty-nine differential metabolites and thirteen microbial biomarkers were identified at different layers. The microbial co-occurrence networks, correlations of dominant microbes with specific metabolites, and physicochemical factors significantly shaping the microbial communities in different layers of HTD were illustrated. This study provides valuable insights into the fermentation mechanisms of different HTD types.

## Highlights

• Different layers of HTD showed significant physicochemical and microbial differentiation from day 8.
• Thirty-nine differential metabolites and thirteen species biomarkers distinguishing different layers were characterized.
• The key driving factors influencing the microbial community of HTD across different layers were identified.

## 1 Introduction

Baijiu is a unique traditional spirit in China with a long production history, rich cultural connotations, and the largest market share among the alcoholic drinks in China. Unlike other globally popular distilled spirits, Baijiu is produced through spontaneous solid-state fermentation using Jiuqu as the starter. Jiuqu plays a crucial role in starch saccharification, alcoholic fermentation, and flavor generation during Baijiu production (Xia et al., 2023). Depending on its production process and physical characteristics, Jiuqu is categorized into Daqu, Xiaoqu, and Fuqu. Among the Baijiu products fermented using different types of Jiuqu, the product using Daqu is the most popular liquor in China. Daqu is usually made from grinded wheat or barley which is spontaneously fermented in an open environment. Based on the maximum temperature achieved during the fermentation process, Daqu is usually classified into three types, namely the low- (45–50 °C), medium- (50–60 °C) and high-temperature (60–70 °C) Daqu (HTD), which are used to produce light-, strong- and sauce (or jiang)- flavor Baijiu, respectively (Huang et al., 2024). Currently, the sauce-flavor Baijiu holds the largest revenue share among the three types of Baijiu in China. In the production of sauce-flavor Baijiu, HTD is used not only as the fermentation starter, but also as a raw material, contributing nearly a half of the raw materials used in the whole fermentation process. Therefore, HTD is crucial to the yield and quality of sauce-flavor Baijiu.

HTD is produced through solid state fermentation using wheat as the raw material. It is made through several steps, including crushing raw wheat grains, mixing the crushed wheat with appropriate proportions of water and powder of matured HTD produced in a previous batch (Muqu), shaping the mixture into bricks, stacking the bricks in a chamber (Qu-room), covering the stack with rice straw for spontaneous fermentation for up to 40 days (Fig. S1), and finally storing the fermented Daqu bricks for 3 to 6 months for maturation. Due to temperature and moisture heterogeneities in different positions within the Daqu stack, three types of Daqu bricks with distinct appearance colors are usually formed at the end of fermentation: white, yellow and black Daqu (Wang et al., 2021). White Daqu is usually formed in the upper or external layer of a Daqu stack, black Daqu is usually found in the middle layer, and yellow Daqu is distributed in other layers (Shi et al., 2022). An appropriate proportion of each type of HTD is used for the fermentation of sauce-flavor Baijiu and different types are thought to play different roles in the fermentation process. Therefore, elucidating the differences in physicochemical properties, metabolites, and microbial communities among different layers of HTD during the fermentation process is crucial for understanding the functions of the three types HTD in the fermentation of sauce-flavor Baijiu.

Previous studies on microorganisms in different types of HTD mostly focused on mature products. For instance, Hou et al. (2024) employed metagenomic sequencing to compare the microbial differences among the three different colored mature HTDs which had been stored for over six months for ripening, revealing functional complementarity among their microbial communities. Similarly, Shi et al. (2022) used amplicon sequencing to compare the microbial composition at the genus level of three different colored mature HTDs, finding that *Oceanobacillus* and *Thermomyces* dominated the microbiota in white Daqu, while *Kroppenstedtia* and *Thermoascus* were abundant in yellow and black Daqu, respectively. However, studies on physiochemical property changes and microbial community successions during the entire fermentation process of HTD are still limited. Yang et al. (2024b) reported significant differences in the dominant microorganisms and their succession across different layers of HTD during fermentation, but only three time points (the 8th, 20th, and 40th day) of the fermentation process were sampled and the taxonomic resolution was limited to the genus level. In recent years, the understanding of Daqu microorganisms has shifted from the genus level to the species level by using the third-generation sequencing technology such as PacBio small-molecule real-time (SMRT) sequencing (Han et al., 2024; Huang et al., 2024). Zhang et al. (2022) utilized SMRT sequencing, gas chromatograph-mass spectrometry (GC–MS) and gas chromatograph-ion mobility spectrometry (GC-IMS) to elucidate the microbial characteristics and metabolite profiles of HTD at different maturation stages. However, the third-generation sequencing technology has rarely been applied to characterize the microbial community compositions and successions in HTD across different layers at different fermentation stages.

In this study, the microbial community compositions at the species level in HTD samples from different layers at different fermentation stages were compared using the PacBio sequencing, targeting the full-length fungal ITS region and the bacterial 16S rRNA gene. Additionally, the physicochemical properties of the Daqu samples and the contents of various metabolites in the samples were simultaneously analyzed. We aimed to elucidate the microbial species diversities and their successions in HTD in different layers at different fermentation stages; the drivers shaping the microbial community assemblies in different colored HTD bricks at the end of fermentation; and the physicochemical and microbiological properties that determine or affect the functions of different types of HTD. The results are valuable for the quality control of HTD and sauce-flavor Baijiu production.

## 2 Materials and methods

### 2.1 Sample collection

HTD samples were collected in August and September 2023 from a sauce-flavor Baijiu company in Zunyi, Guizhou province, China. The Daqu bricks were stacked in the Qu-room in the way of 5 (lays) x 5 (rows) x (55–56) (bricks per row). Samples were collected on days 0, 1, 3, 5, 8, 11, 15, 23, 30, and 40 of the fermentation process. On day 0, three Daqu bricks were randomly selected. At each of the subsequent nine time points, three samples were taken from the upper (layer 1), middle (layer 3), and lower (layer 5) layers of a Daqu stack, respectively. In order to fully represent the spatial heterogeneity, the three samples in each layer were taken from the positions near the door, window, and center of the Qu-room, respectively. The samples were collected at the same positions at every time point. A total of 84 Daqu samples were collected throughout the fermentation process. Each Daqu brick sample was thoroughly powdered and mixed under sterile conditions. One portion of each Daqu sample was immediately analyzed for physicochemical properties, while the other portion was stored at −80 °C for further analysis.

### 2.2 Physicochemical property analyses

The physicochemical properties of the HTD samples collected, including moisture, pH, acidity, saccharification and liquefaction enzyme activities were assessed in triplicate according to the guidelines specified in the industry standard QB/T 4257–2011: General Methods for Daqu Analysis. Saccharification enzyme activity refers to the milligrams of glucose converted from soluble starch by one gram of dry Daqu per hour at 35 °C and pH 4.6 (U, mg/g·h), while liquefaction enzyme activity refers to the grams of starch that can be liquefied by one gram of absolutely dry Daqu per hour under the same conditions (U, g/g·h). Additionally, a digital thermometer (DS 1922 T, Shanghai, China) was embedded in the center of a targeted Daqu brick to measure temperature in real time during fermentation. The various physicochemical indicators of HTD during fermentation were visualized using GraphPad Prism 8.

### 2.3 Determination of free amino acids

Free amino acids in the HTD samples collected was determined following a protocol previously reported in (Asaduzzaman & Chun, 2015) with minor modifications. Specifically, 1 g of Daqu powder was vortexed with 10 mL of pure water for 10 min, stored overnight at 4 °C, and then centrifuged at 10,000 rpm for 3 min. Subsequently, 5 mL of the supernatant was mixed with 5 mL of 10 % sulfosalicylic acid and vortexed for 5 min. After further centrifugation at 12,000 rpm for 5 min, 1 mL of the supernatant was filtered through a 0.22 μm membrane (BKMAM, China) before analysis. Free amino acids were quantified using an amino acid auto analyzer (SYKAM S433D, Germany). The analysis was conducted with a cation exchange separation column LCA K06/Na (4.6 × 150 mm), with column temperatures ranging from 58 °C to 74 °C. The mobile phase consisted of two trisodium citrate solutions (0.12 N and 0.2 N) pumped at a flow rate of 0.45 mL/min. A mixture of indene trione solution and pure water was used as the post-column reagent at a flow rate of 0.7 mL/min. The excitation and emission wavelengths were set at 440 nm and 570 nm, respectively, to detect varying concentrations of free amino acids. The concentrations of various amino acids were calculated according to the National Standard of China GB5009.124–2016: Determination of Amino Acids in Food (People's Republic of China, 2016), using the amino acid standard working solution as a reference. The contents of 17 free amino acids were analyzed using the pheatmap, vegan, ggplot2, and mixOmics packages in R (v 4.3.2).

### 2.4 HPLC and HS-SPME-GC–MS analyses

The contents of sugars, alcohols, and organic acids were analyzed using HPLC (Shimadzu, Kyoto, Japan) according to the method described by Luo et al. (2023). The volatile flavor compounds in Daqu were analyzed following a previously reported protocol (Shi et al., 2022) with minor modifications. Specifically, 50 μL of 2-ethylbutyric acid at a concentration of 1.04 g/L was added as an internal standard. The volatile flavor compounds were determined using an ISQ LT GC–MS system (Thermo Fisher, USA) equipped with a DB-FFAP-Sim column (60 m × 0.25 mm × 0.25 μm). Preliminary identification of the compounds was achieved by matching their retention indices with those in the NIST mass spectral library, followed by verification using the Kovats retention index system. The contents of volatile compounds in the HTD samples were analyzed using the ggplot2, vegan, and mixOmics packages in R (v 4.3.2).

### 2.5 Total DNA extraction, amplification and high throughput sequencing

Total DNA extraction was performed following the modified Sodium Dodecyl Sulfate (SDS) method described by Luo et al. (2023). The microspectrophotometer (Nano-300, China) was used to measure the quality and concentration of the DNA extracted, and the concentration was standardized to 50 ng/μL. The full-length internal transcribed spacer (ITS) region of fungi was amplified using 8-bp barcode-tagged universal primers ITS1 (5′-CTT GGT CAT TTA GAG GAA GTA A-3′) and ITS4 (5′-TCC TCC GCT TAT TGA TAT GC-3′), while the full-length 16S rRNA gene of bacteria was amplified using 8-bp barcode-tagged universal primers 27F (5′-AGA GTT TGA TCM TGG CTC AG-3′) and 1492R (5′-TAC GGY TAC CTT GTT AYG ACT T-3′). The Oligo Analyzer tool was used to evaluate primer hairpin formation and self-dimerization potential. All primers were synthesized by Sangon Biotech Co., Ltd. (Shanghai, China) and purified using ULTRAPAGE. The polymerase chain reaction (PCR) system included 1 μL of genomic DNA, 13.5 μL KOD One PCR Master Mix (Merck KGaA, Germany), 1 μL of 0.1 mol/L forward primer, 1 μL of 0.1 mol/L reverse primer, and 13.5 μL of nuclease-free H_2_O. The PCR reactions were as follows: 98 °C for 2 min; followed by 32 cycles consisting of denaturation at 98 °C for 10 s, annealing at 58 °C for 10 s, and extension at 72 °C for 15 s; and a final extension at 72 °C for 5 min. The PCR amplicons were purified using the Beckman Coulter Agencourt AMPure XP system (Beckman Coulter, The United States), which selectively binds DNA fragments ≥100 bp to paramagnetic beads while effectively removing excess primers, free dNTPs, salt ions, and enzymatic impurities, ultimately yielding highly purified PCR products. Sequencing was conducted using the PacBio Sequel II instrument at Biomarker Technologies Corporation (Beijing, China).

### 2.6 Processing of sequence data

Raw circular consensus sequencing (CCS) reads were obtained for each sample by identifying the different Barcode sequences using Lima software. Primer sequences were removed from all reads using Qiime2 (v2020.2) (Bolyen et al., 2019).Subsequent filtering was performed with usearch11 (https://drive5.com/usearch) to exclude sequences with a length being shorter than 300 bp, ambiguous bases, and a mean quality score < 20 and mononucleotide repeats >8-bp, ensuring the retention of high-quality sequences. DADA2 was then employed to denoise these high-quality sequences into Amplicon Sequencing Variants (ASVs) (Callahan et al., 2017). The chimeric sequences were removed de novo using vsearch (v 2.14) (Rognes et al., 2016). Fungal chimeras were further eliminated using the UNITE CHIME reference database (Nilsson et al., 2019), while bacterial chimeras were removed using the GreenGenes2 (2022.10) database. Non-chimeric sequences were clustered into ASVs using the “unoise3” command with 100 % nucleotide sequence similarity. For species annotation, ASV sequences were compared against the SILVA138 (Quast et al., 2013) and UNITE (2023.05) (Nilsson et al., 2019) databases using a 97 % identity threshold, with non-bacterial and non-fungal sequences excluded. The final ASV table was generated using the “usearch global” command (Rognes et al., 2016).

### 2.7 Statistical analysis

The species composition and diversity analyses of fungi and bacteria in the HTD samples collected were conducted using the ggplot2, vegan, dplyr, and igraph packages in R (v 4.3.2). The species diversity was characterized by the Shannon index, while differences in classification between groups were analyzed via independent one-way analysis of variance (ANOVA) followed by Tukey's honest significant difference test (*P* < 0.05). Hierarchical clustering and Nonmetric Multidimensional Scaling (NMDS) were employed to examine differences in microbial community structure between groups. LEfSe was utilized to identify fungal and bacterial biomarkers of different layers of HTD using online resources (Chen et al., 2022). The Spearman rank correlation analysis was used to assess co-occurrence and co-exclusion relationships between various fungal and bacterial species (Shi et al., 2022).The network structure and its major topological indices were visualized and calculated using Gephi (version 0.9.3), including the positive and negative correlation ratios, average degree, average weighted degree, average path length, clustering coefficient, degree centralization, and modularity. The relationship between physicochemical properties and microbial composition was analyzed through Redundancy Analysis (RDA) using Canoco5 software. The fungal and bacterial species having a relative abundance >0.5 % were selected for correlation analysis with metabolites.

## 3 Results

### 3.1 Physicochemical differences among different layers of HTD during fermentation

**Fig. 1:**
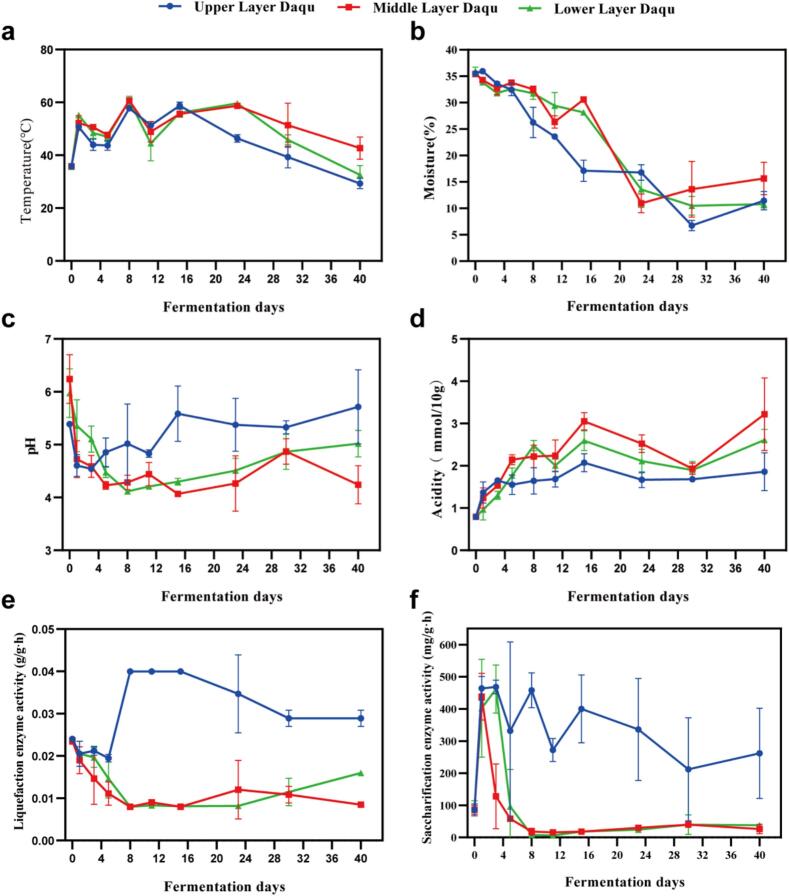
The changes of temperature (a), moisture (b), pH (c), acidity (d), liquefaction enzyme activity (e), and saccharification enzyme activity (f) during the fermentation process of different layers of HTD. Each point represents the average of triplicates ± SE.

Physicochemical properties, including temperature, moisture, pH, saccharification and liquefaction enzyme activities of Daqu bricks in different layers were monitored throughout the whole fermentation process (Fig. 1). The Daqu bricks in the upper layer usually maintained the lowest temperature across nearly all fermentation stages, whereas those in the middle layer exhibited the highest temperatures, especially in the later stage. The temperature difference between the upper and middle layers ranged from 12.1 °C to 13.4 °C during fermentation days 23 to 40 (Fig. 1a). Throughout the fermentation process, the moisture content of Daqu in all layers decreased gradually, with the middle layer retaining the highest moisture content by the end of fermentation (Fig. 1b). During days 5 to 40, the upper layer Daqu exhibited the highest pH and the lowest acidity, while the middle layer Daqu consistently displayed the lowest pH and the highest acidity in nearly all fermentation stages (Fig. 1c, d). During the same fermentation period, the upper layer Daqu exhibited remarkably higher saccharification and liquefaction enzyme activities than those in the other two layers (Fig. 1e, f). The results indicate that the HTD bricks from different layers within the same stack exhibit obvious different physicochemical properties.

### 3.2 Metabolite differences among different layers of HTD during fermentation

The fluctuations in the concentrations of 17 free amino acids throughout the fermentation process were determined. Leucine, glutamic acid, alanine, and proline were the predominant free amino acids in the HTD samples from different layers (Fig. S2a). The Daqu samples from the lower and upper layers exhibited the highest and lowest contents of the total free amino acids, respectively, but the difference was not significant (Fig. S3a). Additionally, we quantitatively analyzed the contents of five organic acids, three sugars, and two alcohols in HTD in different layers during the fermentation process. The results indicated that glucose and lactic acid were the predominant sugar and acid, respectively (Fig. S2b).

**Fig. 2:**
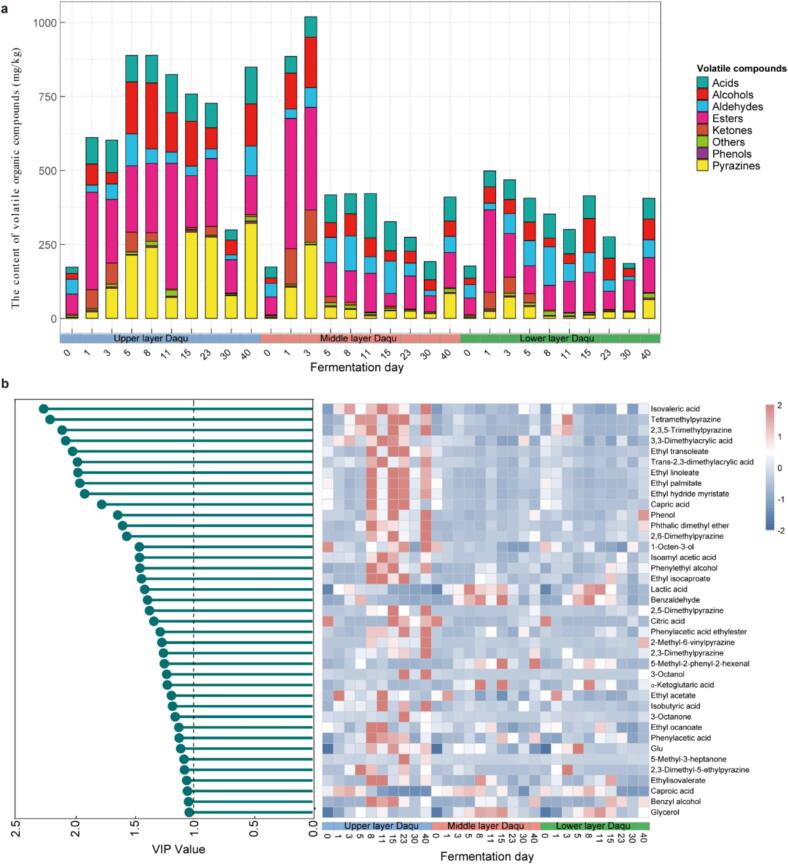
Tempo-spatial dynamics of volatile flavor compounds in HTD during the fermentation process. (a) The relative contents of eight types of volatile flavor compounds during the fermentation process of different layers of HTD. (b) Differential metabolic markers that distinguish different layers of HTD based on VIP values calculated by Partial Least Squares Discriminant Analysis (PLS-DA). The relative contents of 39 metabolites with VIP scores greater than 1.0 are shown in the heatmap according to the *Z*-Score.

A total of 78 volatile metabolites, covering 10 alcohols, 9 pyrazines, 9 esters, 14 acids, 9 aldehydes, 6 ketones, 2 phenols, and 9 other metabolites, were identified in the HTD samples throughout the fermentation process (Fig. S2c). Among the alcohol compounds identified, 2,3-butanediol and 2-tetradecanol exhibited the highest concentrations in the samples from different layers during the initial 0–5 days of fermentation, whereas phenylethanol predominated across samples from different layers during the later stages of fermentation (days 8–40). Among the pyrazine compounds identified, tetramethylpyrazine was the most abundant, with the highest concentrations observed in the upper layer Daqu. The predominant ester, acid, aldehyde, ketone, and phenol were ethyl acetate, isovaleric acid, benzaldehyde, 3-hydroxy-2-butanone, and guaiacol, respectively (Fig. S2c). The contents of the total flavor compounds tested in the upper layer Daqu with different fermentation times were usually higher than those in the other two layers with the same fermentation times, except for the samples in the middle layer during the initial one to three days' fermentation. The middle layer samples showed the higher contents of the total flavor compounds than those in the samples from the upper and lower layers with the same fermentation times. The higher flavor compound contents in the middle layer samples were mainly contributed by pyrazines, esters and alcohols (Fig. 2a). Overall, the content of the total volatile flavor compounds in the upper layer Daqu was significantly higher than those in the other layers throughout the fermentation process, while the flavor compound contents in the lower and middle layer Daqu samples were not significantly different (Fig. S3b).

To identify signature differential metabolites in HTD fermentation across different layers, we performed a variable importance in projection (VIP) analysis based on partial least squares discriminant analysis (PLS-DA). We identified 39 metabolites with VIP scores greater than 1.0 (Table S1), including 35 volatile flavor compounds and lactic acid, citric acid, glutamic acid and glycerol. The concentrations of these metabolites are illustrated in a heatmap, highlighting the distinct variations of them between different layers of HTD during the fermentation process (Fig. 2b). Among the volatile flavor compounds, isovaleric acid (VIP = 2.26), tetramethylpyrazine (VIP = 2.21), and 2,3,5-trimethylpyrazine (VIP = 2.10) have the greatest impact on distinguishing different layers of HTD (Fig. 2b).

### 3.3 Microbial community composition and diversity in different layers of HTD during fermentation

We obtained a total of 854,465 high-quality bacterial 16S rDNA reads and 809,953 high-quality fungal ITS rDNA reads from the 84 HTD samples collected. On average, each sample yielded 10,172 bacterial and 9642 fungal reads. These reads were classified into 1494 bacterial and 444 fungal ASVs, covering 20 phyla, 204 genera, and 311 species of bacteria; and 4 phyla, 106 genera, and 181 species of fungi.

**Fig. 3:**
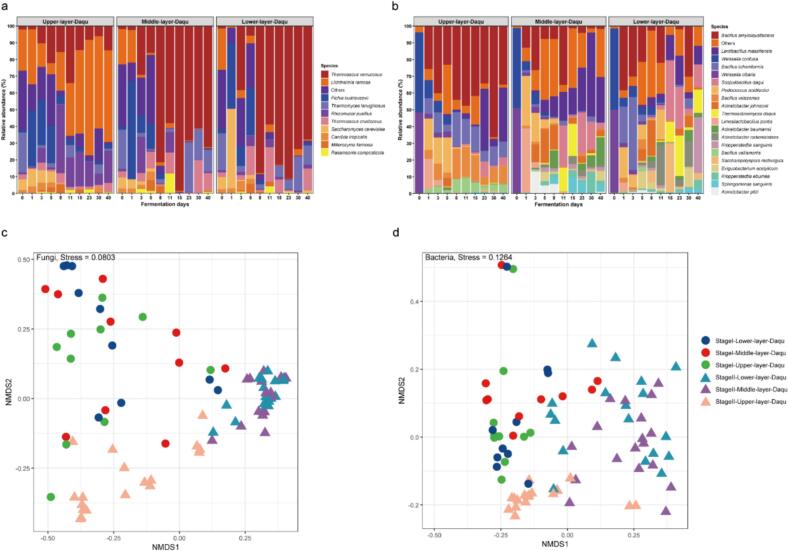
The microbial community differentiation in different layers of HTD during the fermentation process. The fungal (a) and bacterial (b) community compositions at the species level during the fermentation process of different layers of HTD. The species with a relative abundance of less than 0.5 % in all samples were collectively classified as “others”. Nonmetric Multidimensional Scaling analysis (NMDS) of the fungal (c) and bacterial (d) communities during stage I (days 0 to 5) and stage II (days 8 to 40) of fermentation in different layers of HTD.

After normalizing sequence counts to ensure equal sequencing depth in each sample, we conducted comparative analyses of microbial community composition and diversity. For the fungal community, *Lichtheimia ramosa, Thermomyces lanuginosus, Pichia kudriavzevii* and *Saccharomyces cerevisiae* were dominant across different Daqu layers during the fermentation period of days 0 to 5. From days 8 to 40, *Lichtheimia ramosa* and *Rhizomucor pusillus* predominated in the upper layer Daqu, while *Thermoascus verrucosus, Thermoascus crustaceus* and *Thermomyces lanuginosus* were dominant in the middle and lower layers (Fig. 3a). For the bacterial community, *Weissella confusa* and *Weissella cibaria* were the dominant species on day 0. During the fermentation process, *Bacillus amyloliquefaciens*, *Bacillus licheniformis*, *Bacillus velezensis*, and *Bacillus vallismortis* were the predominant bacteria in the upper layer Daqu; while *Bacillus amyloliquefaciens*, *Lentibacillus massiliensis*, *Scopulibacillus daqui*, *Acinetobacter johnsonii* and *Thermoactinomyces daqus* dominated in the middle and lower layers (Fig. 3b). We further analyzed the fungal (Fig. S4a-c) and bacterial (Fig. S4d-f) diversities during the fermentation process of HTD using the Shannon index. During days 0 to 5, the Shannon index of the fungal community in different layers of Daqu decreased on day 1 and then increased, while the bacterial community showed a constant increasing trend. In nearly all the remaining time points, the Shannon index for the fungal community in the upper layer Daqu was consistently higher than that in the middle and lower layers, while the bacterial community Shannon index showed an opposite trend.

To further investigate the differences in microbial community structures among HTD from different layers with different fermentation times, the hierarchical clustering analysis (HCA) was conducted at the species level. The results showed that the fungal composition of HTD from different layers was similar during the 0 to 5 days of fermentation. However, from day 8 to day 40, the fungal composition in the middle and lower layers remained similar, while the upper layer exhibited a distinct profile (Fig. S5a). For the bacterial community, the clustering pattern closely resembled that of the fungi, with the exception of the day 0 samples, which showed a unique bacterial composition (Fig. S5b). Therefore, we divided the entire fermentation process into two stages based on the microbial community differentiation: stage I (fermentation days 0–5, the early stage) and stage II (fermentation days 8–40, the later stage). Subsequently, Non-metric Multidimensional Scaling (NMDS) analysis based on unweighted UniFrac distances was conducted to evaluate differences in microbial community structures in the two fermentation stages and different layers of Daqu. The result showed that in stage I, both the bacterial and fungal communities in different layers were not differentiated, while in stage II, the bacterial and fungal communities in the upper layer were differentiated from those in the middle and lower layers. The overall microbial communities in stages I and II were also clearly different (Fig. 3c-d), supporting further the separation of the two fermentation stages based on the HCA analysis.

### 3.4 Fungal and bacterial biomarkers differentiating different fermentation stages and layers of HTD

**Fig. 4:**
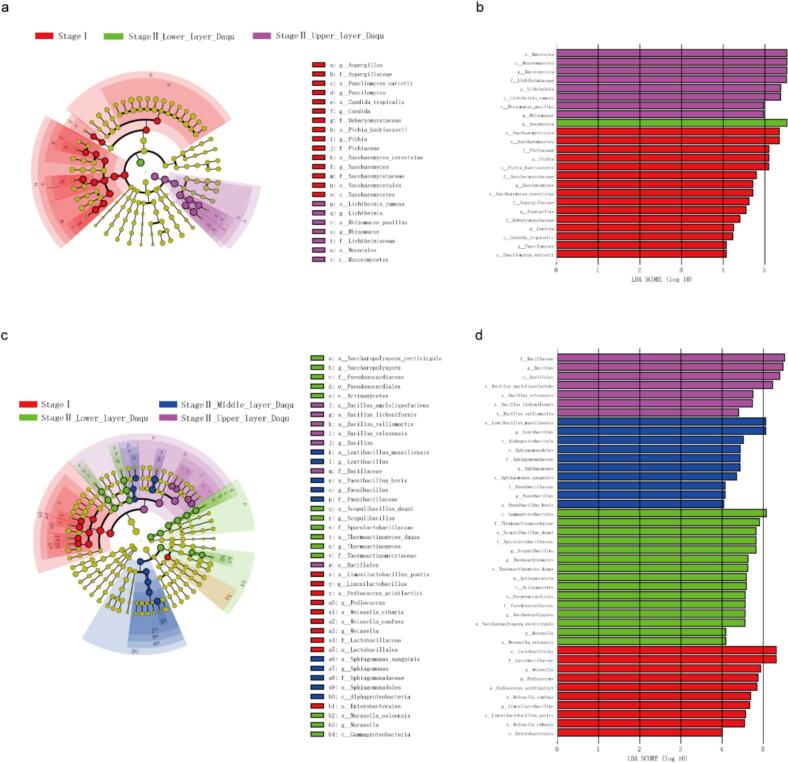
Discriminant microbial taxa between stage I (days 0 to 5) and stage II (days 8 to 40) and between different layers of HTD identified using LEfSe (LDA > 4, *P* < 0.05). The discriminant fungal and bacterial taxa are shown in cladograms (a) and (c), respectively. The significant discriminant taxa for stage I; and upper, middle, and lower layer Daqu in stage II are represented in red, purple, blue, and green, respectively, while the non-discriminant taxa are shown in yellow. Branch areas are shaded according to the variety that ranks highest for that taxon. The LDA (linear discriminant analysis) score reflects the degree of differentiation. Horizontal bar charts display the discriminant fungal (b) and bacterial (d) taxa. (For interpretation of the references to colour in this figure legend, the reader is referred to the web version of this article.)

LEfSe analysis revealed 24 fungal and 42 bacterial taxa, including species and higher taxa, that exhibited significant differences (LDA > 4, *p* < 0.05) in their specific enrichments in stage I and in different layers in stage II (Fig. 4a, c). At the species level, the fungi *Paecilomyces variotii, Candida tropicalis, Pichia kudriavzevii*, and *Saccharomyces cerevisiae*; and the bacteria *Limosilactobacillus pontis, Pediococcus acidilactici, Weissella cibaria,* and *Weissella confusa* were significantly enriched across different layers of Daqu in stage I. In stage II, two fungal species *Lichtheimia ramosa* and *Rhizomucor pusillus* and four bacterial species *Bacillus amyloliquefaciens*, *Bacillus licheniformis*, *Bacillus vallismortis*, and *Bacillus velezensis* were predominantly enriched in the upper layer Daqu; three bacteria species *Lentibacillus massiliensis, Sphingomonas sanguinis* and *Paenibacillus bovis* were significantly enriched in the middle layer Daqu; while four bacterial species *Saccharopolyspora rectivirgula*, *Scopulibacillus daqui*, *Thermoactinomyces daqus*, and *Moraxella osloensis* were significantly enriched in the lower layer Daqu (Fig. 4b, d). These microbes could potentially serve as biomarkers for differentiating the microbial communities of HTD in fermentation stage I and in different layers in stage II.

### 3.5 Microbial interactions and environmental drivers within different layers of HTD during fermentation

**Fig. 5:**
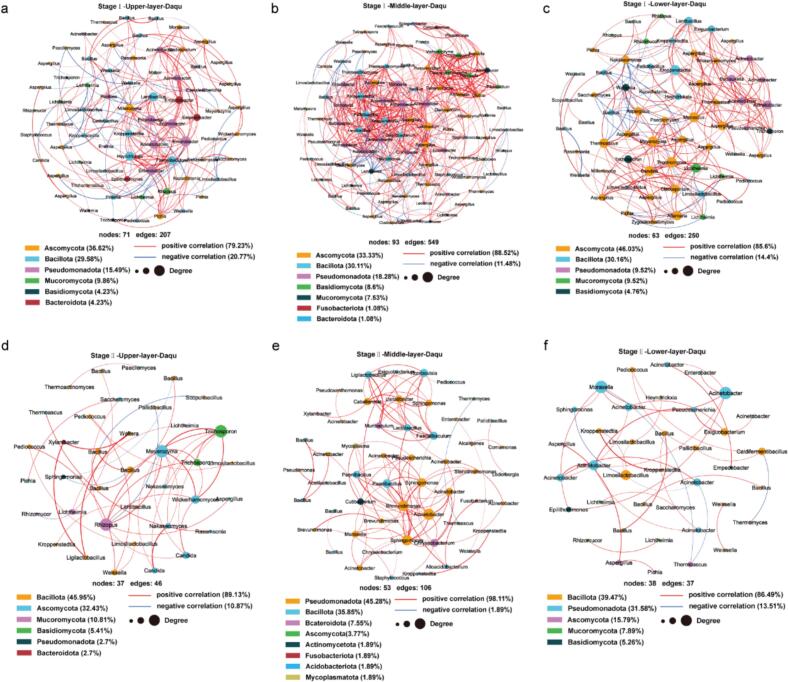
The microbial co-occurrence network structures (*r* > 0.6, *P* < 0.05) in the upper (a), middle (b), and lower (c) layer Daqu during stage I (days 0 to 5); and in the upper (d), middle (e), and lower (f) layer Daqu during stage II (days 8 to 40) of the fermentation process.

Microbial community structure is co-driven by interspecies interactions and environmental conditions. Microbial co-occurrence network analysis, based on the Spearman correlation coefficients of species with a relative abundance greater than 0.1 % (|R| > 0.5, *P* < 0.05), was conducted to elucidate the interactions within the microbial communities of HTD in the two fermentation stages and different layers. Overall, there are significant differences in the microbial co-occurrence networks between stage I and stage II. For the same layer Daqu, stage I had higher number of nodes and edges, average (weighted) degree, and degree centralization but lower positive correlation ratio and modularity compared to stage II (Fig. 5a-f, Table S2). In stage I, positive correlations dominated the microbial co-occurrence networks at different layers, with proportions ranging from 79.23 % to 88.52 % (Fig. 5a-c). The microbial network in the upper layer Daqu exhibited a higher proportion of negative correlations (20.77 %) compared to the middle layer (11.48 %) and the lower layer (14.4 %). The microbial network in the middle layer Daqu had a higher number of nodes and edges, average (weighted) degree, clustering coefficient and degree centralization (Table S2), indicating a more complex structure and stronger interactions within the microbial communities. The microbial co-occurrence network nodes in stage I were predominantly composed of Ascomycota, Bacillota, and Pseudomonadota, which collectively accounted for 81.69 %, 81.72 %, and 85.72 % in the upper, middle, and lower layers, respectively. In stage II, positive correlations also dominated the microbial co-occurrence networks in different layers. The microbial network in the middle layer Daqu also had the highest number of nodes and edges, average (weighted) degree, and clustering coefficient (Table S2), compared to those in the other two layers. In the upper layer, the combined proportion of the most abundant phyla Bacillota and Ascomycota accounted for 78.38 % of the co-occurrence network nodes (Fig. 5d). However, in the middle and lower layers, the sum of the most abundant phyla Pseudomonadota and Bacillota occupied 81.13 % and 71.05 % of the co-occurrence network nodes, respectively (Fig. 5e-f). This indicates that in stage II, the microbial co-occurrence network in the upper layer Daqu was predominantly driven by interactions between bacteria and fungi, while in the middle and lower layers, bacterial interactions were dominant in both layers and stronger in the middle layer.

**Fig. 6:**
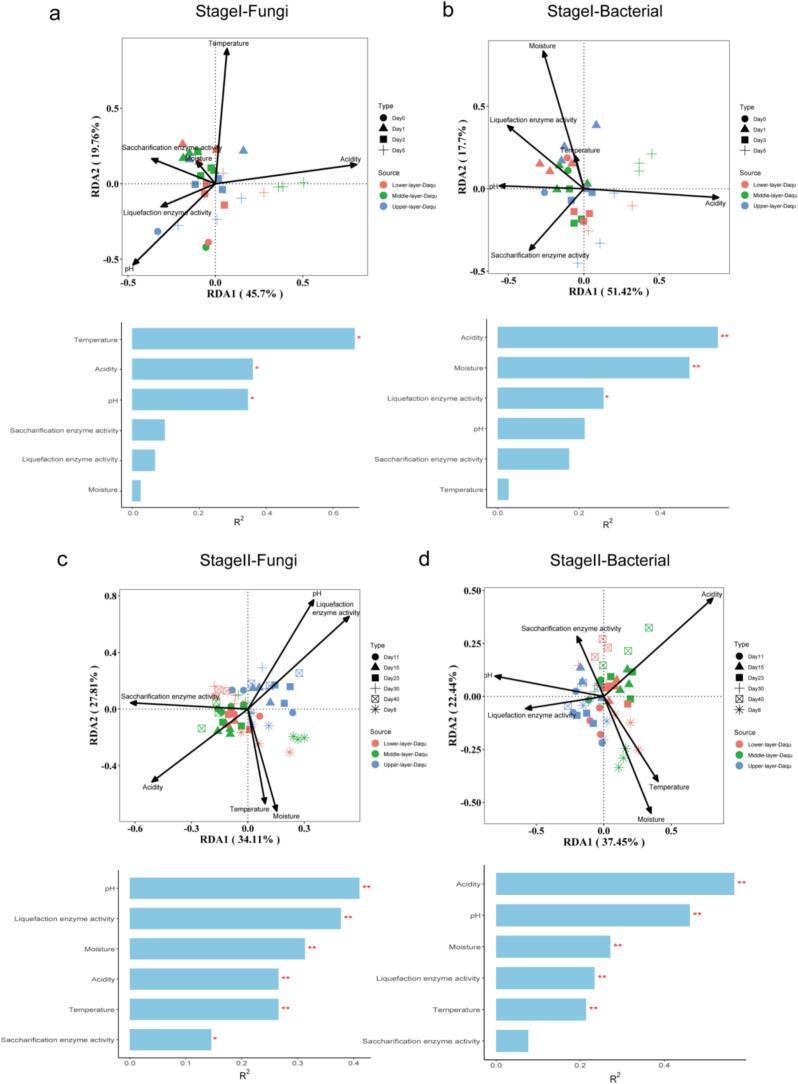
Redundancy analysis (RAD) illustrating the impacts of physicochemical properties on the microbial community compositions in different layers and different fermentation stages of HTD. *, 0.01 < *P* < 0.05; **, 0.001 < *P* < 0.01.

Subsequently, we performed redundancy analysis (RDA) to elucidate the influence of physicochemical properties, including temperature, moisture, acidity, pH, and saccharification and liquefaction enzyme activities, on the microbial community structure within HTD in different fermentation stages and layers. The results showed that the first two ordination axes of the RDA explained 65.46 % and 69.12 % of the variations in the fungal and bacterial community compositions, respectively, in stage I (Fig. 6a, b); and 61.92 % and 59.89 % of the variations in the fungal and bacterial community compositions, respectively, in stage II (Fig. 6c, d). Specifically, in stage I, the formation of the fungal community in different layers of HTD was significantly influenced by temperature, acidity, and pH (Fig. 6a), while the bacterial community was primarily driven by acidity, moisture, and liquefaction enzyme activity (Fig. 6b). In stage II, the formation of microbial communities in different layers of HTD was significantly influenced by five physicochemical factors, with pH and liquefaction enzyme activity primarily affecting the upper layer, while acidity predominantly drove the microbial communities in the middle and lower layers (Fig. 6c-d).

### 3.6 Correlations between microbial community and metabolites during HTD fermentation

**Fig. 7:**
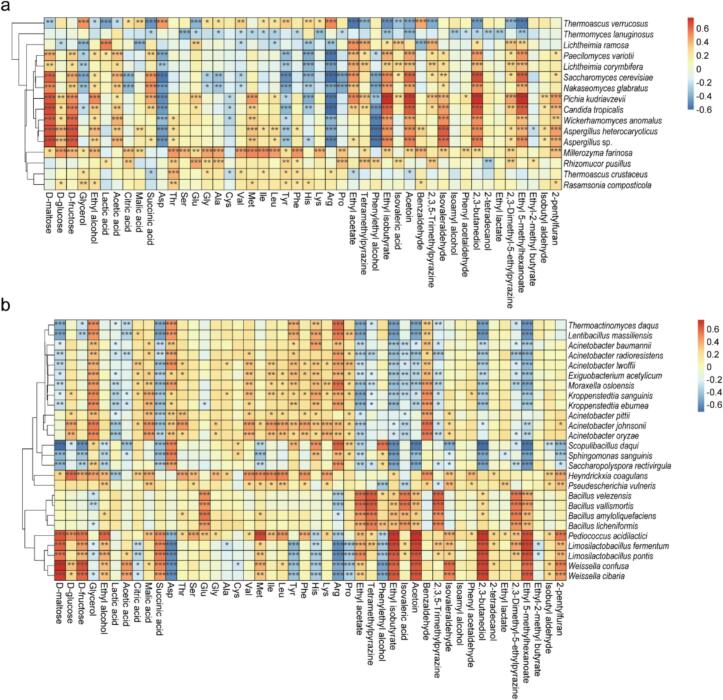
Correlation of fungal (a) and bacterial (b) species with metabolites during the fermentation process of HTD. Heatmaps were constructed based on Spearman correlation coefficients with *P* < 0.05 between microbial species with relative abundance greater than 0.5 % and metabolites. Blue and red represent negative and positive correlation, respectively. *, 0.01 < *P* < 0.05; **, 0.001 < *P* < 0.01; ***, *P* < 0.001. (For interpretation of the references to colour in this figure legend, the reader is referred to the web version of this article.)

The metabolites formed during fermentation are generally produced by microbial metabolic activities. We further analyzed the relationships between the dominant microorganisms (16 fungal and 26 bacterial species) with relative abundances greater than 0.5 % and the key metabolites using Spearman correlation coefficients (Fig. 7a-b). The results revealed that *Paecilomyces variotii*, *Lichtheimia corymbifera*, *Saccharomyces cerevisiae*, *Nakaseomyces glabratus*, *Pichia kudriavzevii*, *Candida tropicalis*, *Wickerhamomyces anomalus*, *Aspergillus heterocaryoticus* and *Aspergillus* sp. similarly had significant positive correlations with the contents of maltose, fructose, acetic acid and five volatile flavor compounds (ethyl isobutyrate, acetoin, isovaleraldehyde, 2,3-butanediol and ethyl 5-methylhexanoate) but significant negative correlations with aspartic acid, tyrosine, and arginine contents (Fig. 7a). *Millerozyma farinosa* showed positive correlations with nearly all amino acids and flavor compounds, whereas *Thermomyces lanuginosus* exhibited the opposite trend. Within the bacterial community, 12 species from the genera *Thermoactinomyces*, *Lentibacillus*, *Acinetobacter*, *Exiguobacterium*, *Moraxella*, and *Kroppenstedtia* exhibited positive correlations with the formations of glycerol, citric acid, malic acid, benzaldehyde, and most free amino acids. Four species within the genus *Bacillus* displayed highly significant positive correlations with the productions of glutamate, ethyl acetate, tetramethylpyrazine, ethyl isobutyrate, isovaleric acid, acetoin, 2,3,5-trimethylpyrazine, 2,3-dimethyl-5-ethylpyrazine, and ethyl 5-methylhexanoate. Additionally, five species from the genus *Pediococcus*, *Weissella*, and *Limosilactobacillus*, belonging to the family *Lactobacillaceae*, exhibited highly significant positive correlations with the contents of maltose, fructose, ethyl alcohol, succinic acid, methionine and seven flavor compounds, including ethyl acetate (Fig. 7b).

## 4 Discussion

This study systematically investigates the physicochemical properties, metabolic profiles, and microbial succession of HTD from both spatial and temporal perspectives. The results reveal that, after an early (the first five days) fermentation stage (stage I), the microbial community structure in the upper layer Daqu differentiated significantly from those in the middle and lower layers during the latter fermentation stage (stage II). The physicochemical factors driving the microbial compositions in the upper and the other layers are distinct. In addition, this study identifies 13 species-level microbial biomarkers and 39 metabolites that differ clearly in different layers of HTD throughout the fermentation process. Microbial co-occurrence network analysis further uncovers layer-specific microbial interactions and highlights key bacterial and fungal taxa that are positively correlated with crucial metabolites. Collectively, this research offers a comprehensive understanding of the spatiotemporal heterogeneity of HTD, providing important insights into the formation mechanisms underlying the three distinct HTD types.

To date, research on HTD has primarily concentrated on its fermentation and post-fermentation processes, without tracking dynamic changes across both spatial and temporal scales. For instance, several studies have investigated microbial communities and physiochemical characteristics of mature HTD with three distinct colors (Shi et al., 2022; Yang et al., 2023), or from different geographical regions (Zhang et al., 2024), or with different qualities (Tang et al., 2024). Other studies have compared temporal microbiome and metabolome dynamics between HTD made by mechanized and traditional processes (Shi et al., 2024; Xing et al., 2025), and the impact of storage time on the microecological properties of HTD (Yang et al., 2024a). However, microbial community composition is influenced by multiple scales (Ladau & Eloe-Fadrosh, 2019). The microbial community of HTD is influenced not only by temporal scales during the fermentation process but also by spatial scales within the same fermentation workshop. Therefore, investigating the diversity and structural characteristics of microbial community in HTD across multiple spatial and temporal scales is essential for achieving a better understanding. Recently, Yang et al. (2024b) employed the gradient internal standard absolute quantification method to perform amplicon sequencing of the 16S rRNA V3-V4 region and the ITS2 region, and to reveal the mechanisms underlying the formation and spatiotemporal differentiation of the microbiota in HTD. While this method enables quantification of microbial abundance, it is based on partial 16S rRNA gene and ITS region and thus unable to achieve an understanding of the spatiotemporal differentiation of the microbiota in HTD at the species level. In contrast, the present study utilizes PacBio SMRT sequencing to obtain full-length bacterial 16S rRNA and fungal ITS sequences, enabling more accurate species-level identification. This approach provides a robust and comprehensive characterization of HTD in terms of microbial diversity, community structure, and environmental drivers across both spatial and temporal dimensions.

The microbial communities in different layers and fermentation stages within the same Qu-room are shaped by distinct physicochemical factors, leading to progressive niche differentiation. Our findings reveal distinct microbial species enrichments in Daqu from different fermentation stages and located in different layers in the latter fermentation stage (Fig. 4a-d). Consistent with previous findings by Yang et al. (2024b), the succession of microbial communities across different Daqu layers is driven by multiple factors, including pH, acidity, temperature, and moisture (Fig. 6a-d). Importantly, bacteria and fungi do not function as isolated units, but instead form dynamic networks shaped by mutualism, competition, and other biotic interactions (Banerjee et al., 2019). Substantial evidence demonstrates that the structural characteristics of these networks, quantified by network topology parameters, effectively reflect the stability of microbial communities (Grilli et al., 2016). Modularity reflects whether microbial species can be grouped into co-occurring clusters. In mature communities, higher modularity indicates stronger functional diversity and greater resistance to disturbance, thereby contributing to community stability (Lurgi et al., 2019). The average degree measures the extent of connections among species, reflecting their connectivity within the network (Zhou et al., 2010). In the first fermentation stage, the microbial co-occurrence network exhibits relatively high average weighted degree (Table S2), suggesting frequent microbial interactions and active information transfer. This facilitates rapid substrate degradation and the initiation of core metabolic pathways. However, the relatively low modularity at this stage indicates that clear functional partitioning of the network has yet to form (Table S2), with the community still undergoing dynamic assembly and selection, resulting in comparatively weaker stability. Moreover, the low proportion of positive correlations among microbes further suggests that the community is primarily in an early phase of competition and niche reorganization (Table S2), with cooperative relationships not yet established. In the second fermentation stage, network modularity increases, and functional partitioning and synergistic interactions among microbes become more pronounced, indicating a trend toward community structural stability. Additionally, the middle layer of Daqu experiences prolonged high-temperature and high-acidity conditions throughout fermentation (Fig. 1a-b), generating strong environmental selection pressures. This promotes the enrichment of dominant microbial groups capable of coexistence and metabolic complementarity. These groups construct tighter metabolic interaction networks, characterized by increased numbers of nodes and edges, higher weighted degree, clustering coefficient, and centralization (Table S2), thereby enhancing microbial community synergism and the stability of functional core structures. This evolution of co-occurrence network topology effectively reflects the trajectory of microbial community functional succession during fermentation and provides theoretical support for understanding the dynamic stability mechanisms of complex micro-ecosystems.

The enzymatic activity of Daqu, primarily conferred by its resident microorganisms, plays a pivotal role in determining the overall quality of Daqu (Han et al., 2023; Huang et al., 2024). Our results indicate that in the second stage of fermentation, the upper layer Daqu exhibits the highest saccharification and liquefaction enzyme activities (Fig. 1e-f). This is consistent with previous findings on white Daqu formed in upper fermentation layers (Shi et al., 2022). The saccharification and liquefaction process of Baijiu fermentation are primarily triggered by Daqu, wherein starch is hydrolyzed into dextrin, maltose, and glucose by various hydrolases. These sugars serve as substrates for microbial growth and metabolism in subsequent alcoholic fermentation. The key enzymes involved in the saccharification and liquefaction process include α-amylase (EC 3.2.1.1) and α-glucosidase (EC 3.2.1.3), both of which have critical implications for Baijiu production (Zeng et al., 2021). Shi et al. (2022) reported that species of the Bacillaceae family dominate the bacterial community in white Daqu, which is consistent with our observations in the second stage of fermentation, where the bacteria in the upper layer Daqu are primarily *Bacillus amyloliquefaciens*, *B. licheniformis*, and *B. velezensis* (Fig. 3b). *Bacillus* species are thermotolerant microorganisms known for producing a wide array of hydrolytic enzymes, including amylase, protease, lipase, cellulase, and glucanase (Wu et al., 2021), which contribute to both starch degradation and flavor compound generation during fermentation (He et al., 2019). For example, *B. amyloliquefaciens* has been reported to produce α-amylase (Lee et al., 2006), while *B. licheniformis* strains isolated from HTD exhibit strong liquefaction enzyme activity (Creusot & Gruppen, 2008). Moreover, He et al. (2019) demonstrated that inoculating Daqu with *B. velezensis* significantly enhanced its liquefaction, saccharification, and esterification capabilities, alongside increased production of alcohols, esters, and pyrazines.

In addition to bacteria, certain molds also contribute to the degradation of macromolecules such as starch during the early fermentation stages, thereby supplying essential enzymatic activity to the fermentation system (Wang et al., 2018). *Lichtheimia ramosa* has been identified as a key liquefaction-associated fungus in the early-stage Daqu (Zhu et al., 2023), and genes encoding α-glucosidase, α-amylase, and saccharification enzymes in HTD are often affiliated with *Rhizopus* species, which are recognized for their strong saccharification capacity (Gan et al., 2019). *Rhizomucor pusillus* is also an important source of saccharification enzymes in both Jiang-flavor and light-flavor Daqu (Li et al., 2015). Consistent with these reports, our results show that *L. ramosa* and *R. pusillus* are dominant fungal taxa in the upper layer Daqu during the second fermentation stage (Fig. 3a). Collectively, these findings underscore the critical role of microbial community composition in driving the enzymatic potential and quality of Daqu.

Physicochemical factors exert distinct influences on microbial communities in Daqu at different fermentation stages (Li et al., 2016). Among these factors, temperature is regarded as a key regulatory factor shaping the succession dynamics of microbial communities (Wang et al., 2011). Yeasts represented by *Saccharomyces cerevisiae* and *Pichia kudriavzevii*, as well as lactic acid bacteria represented by *Weissella confusa*, *Weissella cibaria*, and *Pediococcus acidilactici*, are primarily distributed during the first stage of Daqu fermentation (Fig. 3a-b). These microorganisms thrive at optimal temperatures around 30–37 °C, but their growth is inhibited as fermentation reaches peak temperatures by day eight (Fig. 1a; Fig. 3a-b). At this high-temperature stage, thermophilic or thermotolerant species originating from Muqu become dominant (Liu et al., 2023), including *Thermoascus verrucosus*, *Lichtheimia ramosa*, *Thermomyces lanuginosus*, and *Rhizomucor pusillus* (Fig. 3a). In addition, our results show that the upper layer Daqu exhibits the lowest acidity and highest pH, whereas the middle layer shows the highest acidity and lowest pH (Fig. 1c-d). Lactic acid, the predominant organic acid produced during HTD fermentation (Fig. S2b), is capable of lowering pH to the level that inhibits the growth of most other microorganisms, including common human pathogens (Reddy et al., 2008). Since the upper layer Daqu has the largest contact area with air, the lactic acid bacteria in this layer are unable to produce lactic acid thorough anaerobic fermentation, thus resulting in the lowest acidity. In contrast, the oxygen concentration is the lowest in the middle layer Daqu, promoting the lactic acid bacteria to produce lactic acid through anaerobic fermentation, and thus leading to the highest acidity. Consequently, acid-sensitive Bacillus species become the dominant bacteria in the upper layer Daqu during the second stage fermentation (Fig. 3b). In summary, the oxygen content differences in different layers of the Daqu stack leads to the selection of different survival strategies by microorganisms in each layer during the first stage of fermentation, resulting in the production of different metabolic byproducts. The accumulation of these products alters the microenvironment of Daqu, leading to significant differences in the microbial communities between the layers during the second stage of fermentation.

The long-term practice of HTD production has demonstrated that, by the end of fermentation, white Daqu is mainly distributed in the upper layer of a Daqu stack, black Daqu is concentrated in the middle layer, and yellow Daqu is primarily distributed in the lower layer and other areas. Therefore, understanding the driving factors shaping the microbial community dynamics at different layers of Daqu is crucial for elucidating the mechanisms underlying the formation of the three types of HTD. This study reveals the microbial diversities and community assemblies, physicochemical and metabolic dynamics and key environmental factors driving the microbial community differentiation and succession at different stages and layers of HTD from a spatiotemporal perspective. The results are helpful for the establishment and optimization of a standardized HTD fermentation process by regulating and stabilizing key environmental factors including temperature and ventilation at different fermentation stages, and thus to achieve better quality control of HTD.

## 5 Conclusions

To our knowledge, this is the first study to reveal the tempo-spatial dynamics of microbial community succession at the species level during the fermentation process of HTD. The results showed that during the latter stage of fermentation, physicochemical properties, metabolites, and microbial communities in the upper layer Daqu were significantly differentiated from those in the middle and lower layers. The microbial co-occurrence network in the upper layer Daqu was also distinct from those in the other layers. We further identified differential metabolites and microbial biomarkers distinguishing the upper and other layers of Daqu; the dominant microbes significantly correlated with specific metabolites; and the main driving factors shaping the microbial community assemblies in different fermentation stages and layers of Daqu. The findings of this study provide valuable insights into the mechanisms underlying the formation of the white, black, and yellow HTD in different layers and offer scientific evidence for the effective utilization of different types of HTD in sauce-flavor Baijiu production. The understanding of the microbial mechanism of HTD fermentation is also indispensable for the development of mechanized technology of HTD production, to cope with the challenges of labor shortage and labor cost rising that the current manual operation practice of HTD fermentation is facing.

## CRediT authorship contribution statement

**Yu-Hua Wei:** Writing – review & editing, Writing – original draft, Visualization, Investigation, Data curation, Conceptualization. **Zhang Wen:** Writing – original draft, Methodology, Investigation, Data curation. **Shang-Jie Yao:** Methodology, Data curation. **Ping-Yan Cheng:** Methodology, Data curation. **Wei Huang:** Investigation, Data curation. **Li-Li Jiang:** Investigation, Data curation. **Mei Bai:** Investigation, Data curation. **Da-Yong Han:** Methodology, Data curation. **Liang Song:** Methodology, Data curation. **Hai-Yan Zhu:** Methodology, Data curation. **Feng-Yan Bai:** Writing – review & editing, Validation, Supervision, Conceptualization. **Di-Qiang Wang:** Project administration, Funding acquisition, Conceptualization. **Pei-Jie Han:** Writing – review & editing, Project administration, Funding acquisition, Conceptualization.

## Declaration of competing interest

The authors declare that they have no known competing financial interests or personal relationships that could have appeared to influence the work reported in this paper.

## Data Availability

Data will be made available on request.

## Appendix A Supplementary data

Supplementary material 1 Significant metabolic markers (VIP > 1) across fermentation layers of HTD. Supplementary material 1

Supplementary material 2 Topology parameters of co-occurrence networks. Supplementary material 2

## References

[bb0005] Asaduzzaman A.K.M., Chun B.S. (2015). Recovery of functional materials with thermally stable antioxidative properties in squid muscle hydrolyzates by subcritical water. Journal of Food Science and Technology.

[bb0010] Banerjee S., Walder F., Büchi L., Meyer M., Held A.Y., Gattinger A., van der Heijden M.G.A. (2019). Agricultural intensification reduces microbial network complexity and the abundance of keystone taxa in roots. The ISME Journal.

[bb0015] Bolyen E., Rideout J.R., Dillon M.R., Bokulich N.A., Abnet C.C., Al-Ghalith G.A., Caporaso J.G. (2019). Reproducible, interactive, scalable and extensible microbiome data science using QIIME 2. Nature Biotechnology.

[bb0020] Callahan B.J., McMurdie P.J., Holmes S.P. (2017). Exact sequence variants should replace operational taxonomic units in marker-gene data analysis. The ISME Journal.

[bb0025] Chen T., Liu Y.X., Huang L. (2022). ImageGP: An easy-to-use data visualization web server for scientific researchers. iMeta.

[bb0035] Creusot N., Gruppen H. (2008). Hydrolysis of whey protein isolate with *Bacillus licheniformis* protease: Aggregating capacities of peptide fractions. Journal of Agricultural and Food Chemistry.

[bb0040] Gan S.H., Yang F., Sahu S.K., Luo R.Y., Liao S.L., Wang H.Y., Liu H. (2019). Deciphering the composition and functional profile of the microbial communities in Chinese Moutai liquor starters. Frontiers in Microbiology.

[bb0045] Grilli J., Rogers T., Allesina S. (2016). Modularity and stability in ecological communities. Nature Communications.

[bb0050] Han P.-J., Song L., Wen Z., Zhu H.-Y., Wei Y.-H., Wang J.-W., Bai M., Luo L.-J., Wang J.-W., Chen S.-X., You X.-L., Han D.-Y., Bai F.-Y. (2024). Species-level understanding of the bacterial community in Daqu based on full-length 16S rRNA gene sequences. Food Microbiology.

[bb0055] He G., Huang J., Zhou R., Wu C., Jin Y. (2019). Effect of fortified Daqu on the microbial community and flavor in Chinese strong-flavor liquor brewing process. Frontiers in Microbiology.

[bb0060] Hou Q., Wang Y., Qu D., Zhao H., Tian L., Zhou J., Liu J., Guo Z. (2024). Microbial communities, functional, and flavor differences among three different-colored high-temperature Daqu: A comprehensive metagenomic, physicochemical, and electronic sensory analysis. Food Research International.

[bb0065] Huang Y., Li D., Mu Y., Zhu Z., Wu Y., Qi Q., Mu Y., Su W. (2024). Exploring the heterogeneity of community and function and correspondence of “species-enzymes” among three types of Daqu with different fermentation peak-temperature via high-throughput sequencing and metagenomics. Food Research International.

[bb0070] Ladau J., Eloe-Fadrosh E.A. (2019). Spatial, temporal, and phylogenetic scales of microbial ecology. Trends in Microbiology.

[bb0075] Lee S., Mouri Y., Minoda M., Oneda H., Inouye K. (2006). Comparison of the wild-type α-amylase and its variant enzymes in *Bacillus amyloliquefaciens* in activity and thermal stability, and insights into engineering the thermal stability of *Bacillus* α-amylase. Journal of Biochemistry.

[bb0080] Li P., Lin W., Liu X., Wang X., Luo L. (2016). Environmental factors affecting microbiota dynamics during traditional solid-state fermentation of Chinese Daqu starter. Frontiers in Microbiology.

[bb0085] Li Z., Chen L., Bai Z., Wang D., Gao L., Hui B. (2015). Cultivable bacterial diversity and amylase production in two typical light-flavor Daqus of Chinese spirits. Frontiers in Life Science.

[bb0090] Liu S., Zhou Y., Ma D., Zhang S., Dong Y., Zhang X., Mao J. (2023). Environment microorganism and mature Daqu powder shaped microbial community formation in mechanically strong-flavor Daqu. Food Bioscience.

[bb0095] Luo L.J., Song L., Han Y., Zhen P., Han D.Y., Zhao X., Bai F.Y. (2023). Microbial communities and their correlation with flavor compound formation during the mechanized production of light-flavor baijiu. Food Research International.

[bb0100] Lurgi M., Thomas T., Wemheuer B., Webster N.S., Montoya J.M. (2019). Modularity and predicted functions of the global sponge-microbiome network. Nature Communications.

[bb0105] Nilsson R.H., Larsson K.H., Taylor A.F.S., Bengtsson-Palme J., Jeppesen T.S., Schigel D., Abarenkov K. (2019). The UNITE database for molecular identification of fungi: Handling dark taxa and parallel taxonomic classifications. Nucleic Acids Research.

[bb0110] People's Republic of China (2016).

[bb0115] Quast C., Pruesse E., Yilmaz P., Gerken J., Schweer T., Yarza P., Glöckner F.O. (2013). The SILVA ribosomal RNA gene database project: Improved data processing and web-based tools. Nucleic Acids Research.

[bb0120] Reddy G., Altaf M., Naveena B.J., Venkateshwar M., Kumar E.V. (2008). Amylolytic bacterial lactic acid fermentation - a review. Biotechnology Advances.

[bb0125] Rognes T., Flouri T., Nichols B., Quince C., Mahé F. (2016). VSEARCH: A versatile open source tool for metagenomics. PeerJ.

[bb0130] Shi G., Fang C., Xing S., Guo Y., Li X., Han X., Lin L., Zhang C. (2024). Heterogenetic mechanism in high-temperature Daqu fermentation by traditional craft and mechanical craft: From microbial assembly patterns to metabolism phenotypes. Food Research International.

[bb0135] Shi W., Chai L.J., Fang G.Y., Mei J.L., Lu Z.M., Zhang X.J., Xu Z.H. (2022). Spatial heterogeneity of the microbiome and metabolome profiles of high-temperature Daqu in the same workshop. Food Research International.

[bb0140] Tang P., Wang L., zhao, Q., Lu, J., Qiao, M., Li, C., Xiao, D., & Guo, X. (2024). Characterization of key aroma compounds and relationship between aroma compounds and sensory attributes in different quality of high temperature Daqu. LWT.

[bb0145] Wang H.Y., Gao Y.B., Fan Q.W., Xu Y. (2011). Characterization and comparison of microbial community of different typical Chinese liquor Daqus by PCR-DGGE. Letters in Applied Microbiology.

[bb0150] Wang X.D., Ban S.D., Qiu S.Y. (2018). Analysis of the mould microbiome and exogenous enzyme production in Moutai-flavor Daqu. Journal of the Institute of Brewing.

[bb0155] Wang Y., Cai W., Wang W., Shu N., Zhang Z., Hou Q., Shan C., Guo Z. (2021). Analysis of microbial diversity and functional differences in different types of high-temperature Daqu. Food Science and Nutrition.

[bb0160] Wu X., Jiang Q., Wang Z., Xu Y., Chen W., Sun J., Liu Y. (2021). Diversity, enzyme production and antibacterial activity of *Bacillus* resource in sesame-flavored liquor Daqu..

[bb0165] Xia Y., Luo H., Wu Z., Zhang W. (2023). Microbial diversity in Jiuqu and its fermentation features: Saccharification, alcohol fermentation and flavors generation. Applied Microbiology and Biotechnology.

[bb0170] Xing S., Shi G., Lu J., Fang C., Li C., Yuan S., Shi F., Lin L., Zhang C. (2025). The discrepancy in amino acids within high-temperature Daqu: A novel metabolic marker for the quality evaluation of Daqu. Food Chemistry.

[bb0175] Yang L., Fan W., Xu Y. (2023). Chameleon-like microbes promote microecological differentiation of Daqu. Food Microbiology.

[bb0180] Yang L., Fan W., Xu Y. (2024). Effects of storage period and season on the microecological characteristics of Jiangxiangxing high-temperature Daqu. Food Research International.

[bb0185] Yang L., Fan W., Xu Y. (2024). Qu-omics elucidates the formation and spatio-temporal differentiation mechanism underlying the microecology of high temperature Daqu. Food Chemistry.

[bb0190] Zeng C., Tagawa Y., Yoshizaki Y., Wang T., Yamaguchi M., Kadooka C., Okutsu K., Futagami T., Tamaki H., Takamine K. (2021). The expression profiles of acid-stable α-amylase and acid-labile α-amylase of *aspergillus luchuensis* Mut. Kawachii effect on the microstructure of koji and alcohol fermentation. LWT.

[bb0195] Zhang Y., Xu J., Jiang Y., Niu J., Chen X., Han B.Z. (2022). Microbial characteristics and metabolite profiles of high-temperature Daqu in different maturation stages. World Journal of Microbiology and Biotechnology.

[bb0200] Zhang Z., Meng Y., Wang Y., Hou Q., Zhang H., Zhang M., Hu G., Zhou Y., Pan Q., Guo Z. (2024). Understanding the factors influencing high-temperature Daqu from different geographical regions. Food Bioscience.

[bb0205] Zhou J., Deng Y., Luo F., He Z., Tu Q., Zhi X. (2010). Functional molecular ecological networks. MBio.

[bb0210] Zhu Q., Chen L., Peng Z., Zhang Q., Huang W., Yang F., Du G., Zhang J., Wang L. (2023). The differences in carbohydrate utilization ability between six rounds of sauce-flavor Daqu. Food Research International.

