# Supplementary material for: Tempo-spatial dynamics of physicochemical properties and microbial communities in high-temperature Daqu during the fermentation process
Source: Food Chem X. 2025 Jul 20;29:102815. doi: 10.1016/j.fochx.2025.102815 (PMC12305732; doi:10.1016/j.fochx.2025.102815)
Supplement: Supplementary material 1 — Significant metabolic markers (VIP > 1) across fermentation layers of HTD. [file mmc1.docx]

**Supplementary materials**


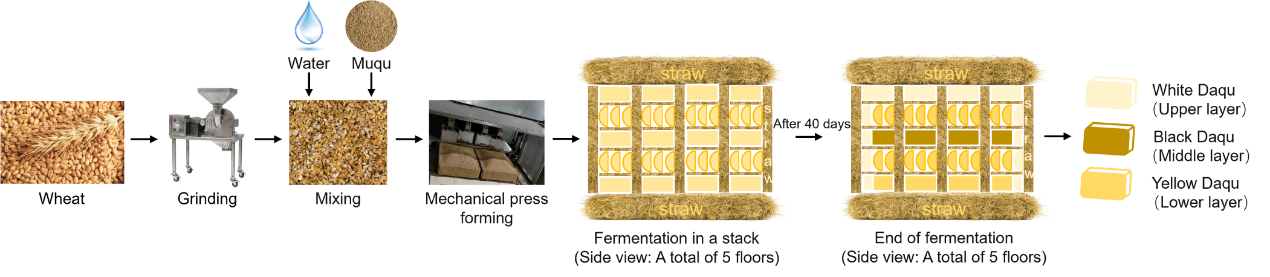


**Figure S1.** The work flow of traditional high temperature Daqu production and the distribution of different types of HTD in a fermentation workshop at the end of fermentation.


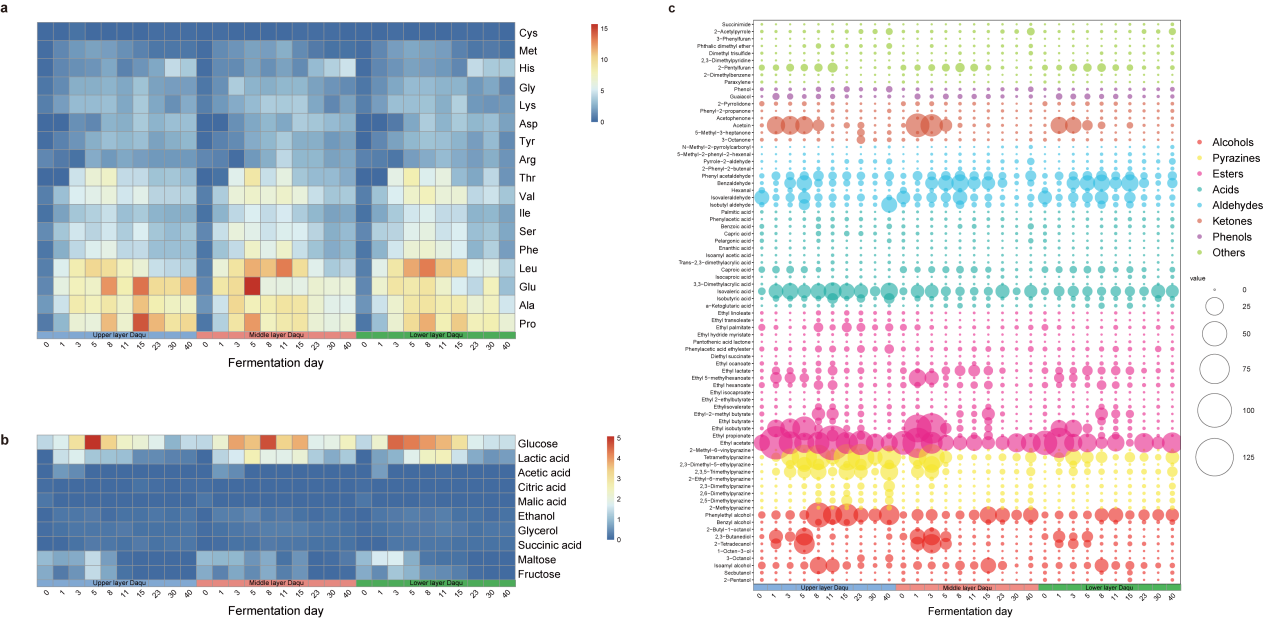


**Figure S2.** Dynamics of the contents of (a) free amino acids (mg/100g), (b) organic acids, sugars, and alcohols (g/100g), and (c) volatile flavor compounds (mg/kg) in different layers of high-temperature Daqu (HTD) during fermentation.


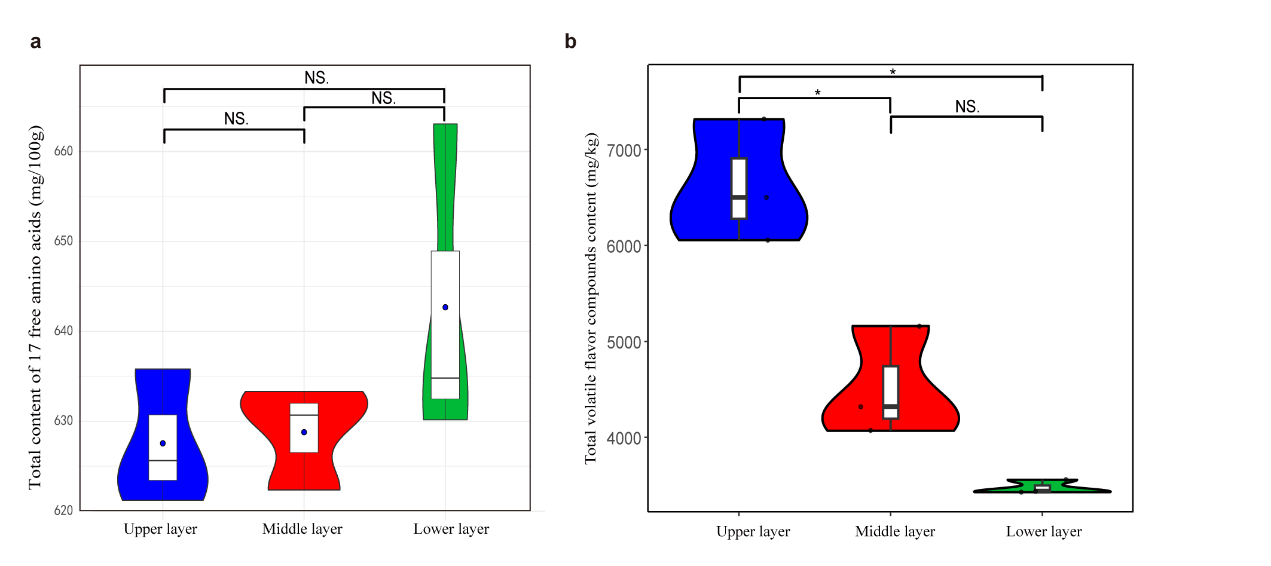


**Figure S3.** The differences of total free amino acids (a) and total volatile flavor compounds (b) in different layers of HTD. *, *P* < 0.05; NS, not significant.


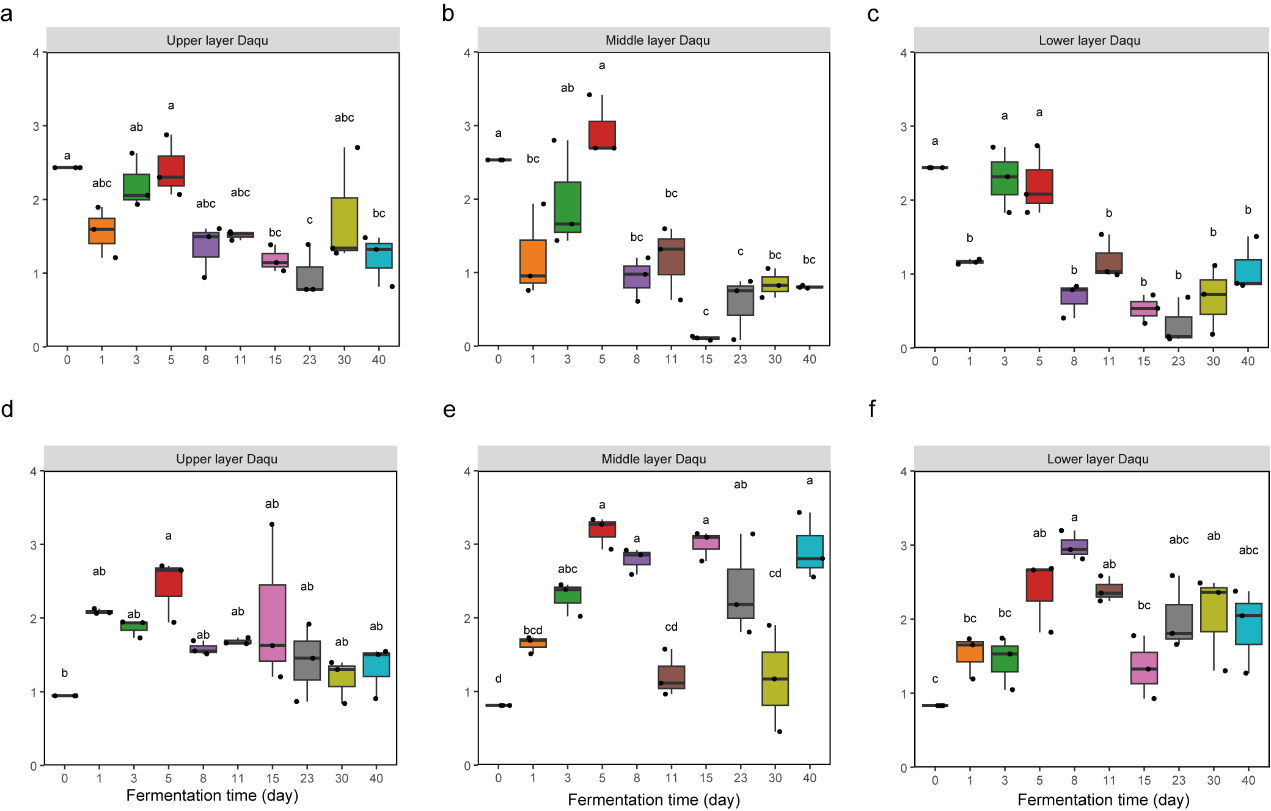


**Figure S4.** Diversities of the fungal (a-c) and bacterial (d-e) communities in terms of Shannon index in different layers of HTD during the fermentation process. Significant differences are denoted by distinct lowercase letters (*P* < 0.05).


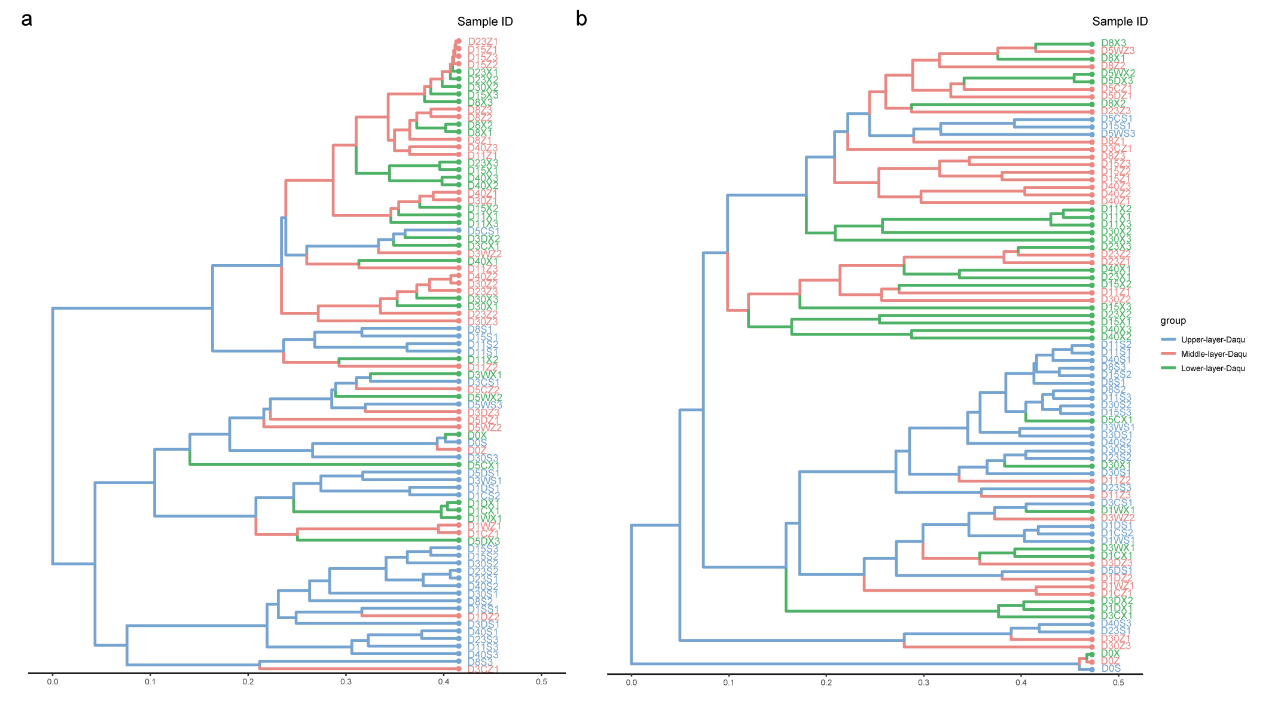


**Figure S5.** Hierarchical cluster analysis based on the fungal (a) and bacterial (b) communities. Meanings of letters in sample ID (D, fermentation day; S, upper layer Daqu; Z, middle layer Daqu; X, lower layer Daqu).
